# OLDER PEOPLE'S ACTIVITY PARTICIPATION (OPAP): FACTOR STRUCTURE AND PSYCHOMETRIC PROPERTIES OF A SCALE

**DOI:** 10.1093/geroni/igac059.884

**Published:** 2022-12-20

**Authors:** Daniel Rong Yao Gan, Yuxin Cao, Wen Liu, John Chye Fung, Tze Pin Ng

**Affiliations:** Simon Fraser University, Vancouver, British Columbia, Canada; National University of Singapore, Singapore, Singapore; University of Iowa, Iowa City, Iowa, United States; National University of Singapore, Singapore, Singapore; National University of Singapore, Singapore, Singapore

## Abstract

Older people’s participation in activities is critical to their health and well-being. Active lifestyle in old age reduces the risk of mortality, prevents chronic diseases, promotes physical and mental wellbeing, and is conducive to active and healthy aging. While various measures of participation have been proposed, a holistic measure that includes both social and individual activity participation is currently unavailable. To enable lifestyle medicine recommendations, we developed the Older People’s Activity Participation (OPAP) scale to understand the constituent factors of older people’s everyday activities. This study examined OPAP’s internal consistency using Cronbach’s alpha, convergent validity using regression analysis, as well as factor structure using exploratory and confirmatory factor analyses in a dense urban setting. Preliminary items assessed engagement in 27 health-related, fitness, recreational, social, productive, and cognitive activities, and were administered to 270 community-dwelling adults aged 50 and older in Singapore. The 17-item OPAP showed acceptable internal consistency (Cronbach’s alpha= .69), and demonstrated convergence with a validated measure of social cohesion (B=.27, p<.001) and Instrumental Activities of Daily Living (B=.32, p<.001). It has a 5-factor structure, namely socializing (alpha=.63), physical training (alpha=.56), listening (alpha=.69), home-making (alpha=.59), and outing (alpha=.58). Acceptable model fit was obtained (RMSEA=0.48, SRMR=0.06, CFI=.90). The OPAP scale is valid and reliable to assess activity participation in Asian older adults. It can be a useful tool to understand older people’s everyday life and lifestyles, relationships between activity participation and health outcomes, and further guide the development and use of effective interventions to promote active and healthy aging.

